# Assessment of Zero-Shot Large Language Model (LLM) Assisted Clinical Trial Matching Processes: A Metastatic Cancer Use Case

**DOI:** 10.64898/2026.07.06.26354647

**Published:** 2026-07-10

**Authors:** Yingjie Weng, Himani Yalamaddi, Danning Fu, Ankita Mishra, Bryan J. Bunning, Andrew B. Martin, Jessica Hope, Vivek Charu, Allison Kurian, Manisha Desai

**Affiliations:** 1 Quantitative Sciences Unit, Stanford University School of Medicine, Palo Alto, CA; 2 Department of Pathology, Stanford University School of Medicine, Stanford, CA; 3 Stanford University School of Medicine, Stanford, CA; 4 Stanford Department of Biomedical Data Science, Stanford, CA

**Keywords:** clinical trial matching, large language models, cancer care

## Abstract

**Introduction:**

For oncology patients with limited treatment options, clinical trials may be a critical lifesaving pathway. Identifying relevant trials, however, is a time-consuming and difficult task. Several patient-trial matching processes incorporating large language models (LLMs) have been proposed to alleviate the burden on patients and oncologists. We aim to explore the benefits and practical challenges of zero-shot LLM-assisted trial matching processes by analyzing the results for a single pancreatic cancer patient.

**Materials and Methods:**

The results of a simple zero-shot LLM-assisted clinical trial matching process for our patient were compared to those of a “human benchmark,” which was developed manually by two of the authors interfacing directly with ClinicalTrials.gov. Performance metrics – sensitivity, specificity, precision, and accuracy – were calculated. In addition, a qualitative content analysis (QCA) of LLM reasoning text was done to identify patterns in “errors,” which we define as a human-LLM discrepancy in final patient eligibility. Implications and severity of errors are discussed.

**Results:**

The zero-shot LLM-assisted process returned potential trials with a sensitivity, specificity, and precision of 81.1%, 89.3%, and 86.5% respectively compared to the human benchmark. Qualitative error analyses revealed that about 73% of errors could potentially be alleviated with improved prompting and information access. Overall performance seemed comparable to that of human reviewers.

**Conclusion:**

The results from this preliminary real-world case study provide additional evidence to the literature in support of the integration of LLMs in clinical trial matching to provide benefit to patients with metastatic cancer with limited options.

## BACKGROUND AND SIGNIFICANCE

### Overview

Participation in a clinical trial for many oncology patients can be transformative and potentially lifesaving. This is especially true when an effective standard-of-care has not been established – as is often the case with complex disease or metastatic cancer – or when a first-line therapy has failed[1,2]. Despite demonstrated benefits, a 2023 estimate posited that only about 7% of adult cancer patients participated in a treatment trial[3]. Low enrollment could be attributed to the onerous nature of identifying an appropriate and accessible trial for a specific patient (which we will refer to as “patient-trial matching”)[4]. The sheer volume of available trials and their unstructured, disease-specific eligibility criteria make the task extremely time-consuming, often precluding patient-trial matching from an oncologist’s typical workflow[5]. Thus, the challenging task of locating and joining a clinical trial is often left to the patient, who likely lacks the time, resources, medical literacy, and understanding of the clinical landscape needed to determine which trials are possibilities for them[4].

### Proposed Methods

ClinicalTrials.gov[6] – an online registry of clinical research studies and results maintained by the National Library of Medicine – offers one of the most comprehensive listings of ongoing U.S. clinical trials. However, these large, ever-expanding databases provide only minimal filtering tools, and their complex interfaces and specialized medical terminology can make it challenging for patients to navigate on their own. Seeking to address the overwhelming volume of trials to review, several solutions have emerged. For example, many online patient-trial matching platforms feature questionnaires (of varying quality and detail) and sometimes employ algorithms to improve matches[7-13]. Some cancer organizations vet disease-specific trials and provide similar questionnaire-based matching tools for patients to locate them[14-16]. In our investigations, however, these questionnaires often did not sufficiently account for non-standardized patient characteristics such as molecular mutations, disease progression, or treatment histories.

LLMs, however, are well-suited to addressing these limitations, excelling at processing large amounts of unstructured, text-based information. As a result, several LLM-based systems have been proposed and implemented for both patient-to-trial matching and trial-to-patient matching (assisting clinical trialists with recruitment, which we will not address here). These systems have been quite successful regardless of the underlying LLM. However, proprietary frontier models (such as the GPT-4 series) tend to perform better – achieving strong patient-to-trial matching metrics, with accuracy ranging from 70%-90% using GPT-4 family models [17,18]. Evaluations of these systems have largely emphasized quantitative performance metrics using synthetic cases, and few have provided detailed case studies or error analyses involving real-world patients. In addition, these systems rarely compared performance to a zero-shot ‘base’ performance of these models without pre-processing of patient or trial eligibility criteria.

In this work, we aimed to contribute to this growing body of research by publishing the performance of a ‘base’ zero-shot LLM-assisted process applied to a real-world use case of metastatic cancer, with the novel inclusion of qualitative content analysis of LLM reasoning text. Using both quantitative and qualitative methods allowed us to: (1) elucidate the strengths and weaknesses of LLM-assisted vs human reasoning in identifying appropriate trials; (2) pinpoint remaining gaps; and (3) provide insights into approaches for improving the next-generation of the clinical trial matching pipeline.

Specifically, we conducted a quantitative evaluation of sensitivity, specificity, precision, and accuracy by comparing patient-clinical trials matching decisions from an LLM-assisted process with our manual human benchmark. We then conducted a qualitative content analysis (QCA) to further evaluate the reasoning texts for the trials with discrepant decisions made by the LLM-assisted process and human benchmark. Qualitative content analysis (QCA), as a systematic method for interpreting text by coding it into themes that capture shared meanings and contextual nuance, has been used in medical AI (artificial intelligence) research to characterize error types in areas such as hallucinations[19], multilingual coding[20], and clinical note extraction[21]. This makes QCA well-suited for analyzing errors in clinical trial matching because both LLM reasoning and assessor decisions can be examined as text. Yet, it has rarely been used to build meaningful benchmarks.

## MATERIALS AND METHODS

### Evaluation process

**Figure 1.**
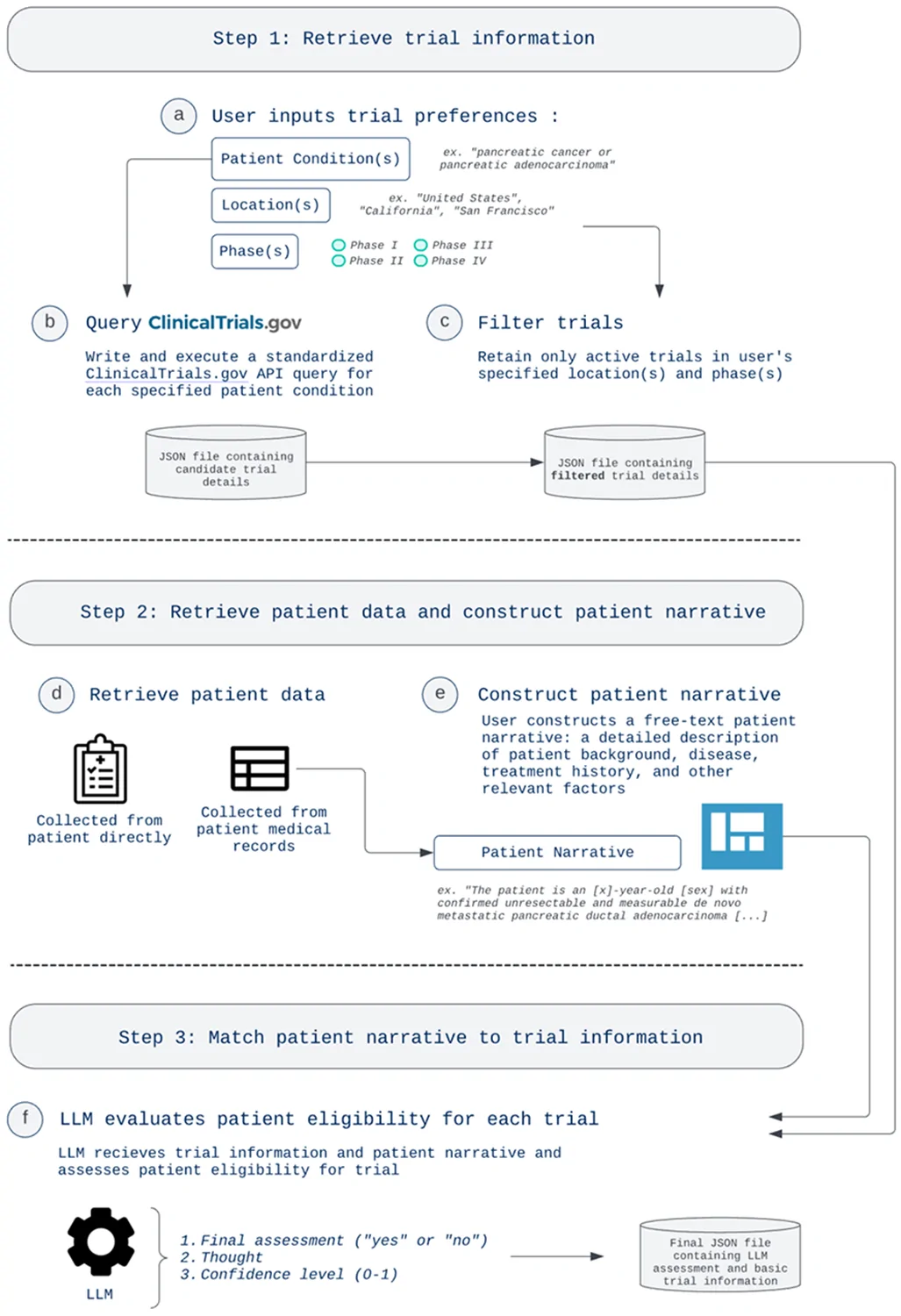
The tested clinical trial matching process

We used a simple zero-shot LLM-assisted patient-to-trial matching process to conduct our analysis. It consisted of the following three steps, broadly similar to recent approaches in the field[22]: 1) retrieval of trial data, 2) retrieval of patient data (from the healthcare system or the patient) and interpretation of patient data into a patient narrative and 3) application of the matching algorithm. The ClinicalTrials.gov (v2) API was used to identify and download initial relevant trial information. The OpenAI Python API Library was used to access the large language models, which were used to match candidate trials to patient information (Supplementary Materials Appendix II). The details of the process are described below and in Figure 1.

#### Step 1. Retrieval of trial data: identify candidate trials for our use case

The search conditions “Solid Tumors,” “Pancreatic Cancer,” and “Pancreatic Adenocarcinoma” were chosen by the authors to retrieve a breadth of potentially relevant trials. The ClinicalTrials.gov API was queried with the terms, and the most relevant and dense trial information (“Brief Title”, “Brief Summary”, and “Eligibility Criteria”) were retrieved and saved as free text. We acknowledged this as a limitation; as the costs of using LLMs decrease, all available trial information should be used. Logistical preferences (the phase of a trial (*multiselect boxes*) and geographic location(s) (*free text*)) were chosen, and only trials that matched these selected preferences were kept for further evaluation.

#### Step 2. Retrieval of patient data to construct a patient narrative

The patient narrative is a free-text, detailed description detailing key patient characteristics (e.g. background, disease, and treatment history) constructed by a user familiar with the patient’s case, similar to a patient vignette. An anonymized version of our patient’s narrative is reproduced below.

**Table:** 

“The patient is a [*redacted age*]-year-old [*redacted sex*] with confirmed unresectable and measurable de novo metastatic pancreatic ductal adenocarcinoma. The tumor is MSI-High negative.
The patient previously failed (progressed on) one regimen of FOLFIRINOX and has received no other treatment.
The patient received comprehensive genetic testing, which revealed the following mutations: KRAS (G12D), TP53 (Y235 and M237 deletion), CDKN2A (T18fs), SMAD4 (A451P), and NTRK3 (V640V).
The patient has confirmed their willingness and ability to comply with study procedures.”

#### Step 3. Application of the matching algorithm

The patient narrative was then inserted into the prompt of the underlying LLM (GPT-4 Turbo) to return an evaluation of the patient’s eligibility to participate in each of the filtered trials from Step 1, including: 1) a binary (“yes” or “no”) adjudication of a patient’s eligibility to participate in each trial, 2) a free text “thought process,” in which the LLM explains its decision-making process, and 3) a confidence level (range from 0 to 1, with higher number indicated greater confidence), intended to test a simple ranking system for trials based on the LLM self-rating its own judgements (not addressed in the current work).

### Quantitative Analysis: Performance metrics for Human-LLM comparison

One of the authors (H.Y.) familiar with patient-trial matching served as our human rater. A second reviewer (Y.W.) independently conducted the assessment, who convened the primary human rater to discuss discrepancies and reach consensus on discrepant findings. In total, 207 candidate trials retrieved from ClinicalTrials.gov were evaluated against our patient case using a “yes”/”no” binary adjudication, along with an explanation and self-rated confidence level, mirroring LLM output. This serves as the “human benchmark” for evaluating LLM’s patient-to-trial matching performance metrics: accuracy, precision, sensitivity, and specificity. While minimal study information (“Brief Title”, “Brief Summary”, and “Eligibility Criteria”) was included in the prompt given to the LLM, the human evaluators were provided the full webpage from ClinicalTrials.gov, which can include additional sections (“Detailed Description” and “Conditions”).

### Qualitative analysis: Qualitative Content Analysis of the Reasoning Texts from LLM and Human Evaluators

In cases where LLM and the human benchmark differed (defined here as an “error”), a qualitative content analysis (QCA) of human and LLM “thought process” text was performed to identify patterns in reasoning errors. Text was coded inductively (where codes and themes are developed a posteriori from the text itself). After codes were developed, each error was independently categorized into one of six “error types” (Table 1.) in two rounds: first by H.Y., and then by Y.W. Discordant results were discussed and resolved.

## RESULTS

For our use case, we identified a total of 207 phase I/II, II, II/III, and IV interventional trials with at least one location in California, United States, that were recruiting, not yet recruiting, available, or enrolling by invitation at the time, as published on ClinicalTrials.gov.

### Quantitative analysis: Performance metrics for Human-LLM comparison

Of the 207 candidate trials, the LLM determined that our patient would be eligible for 89 (yield: 43.0%). Of the 89 trials identified by the LLM, 77 were considered matches by the human reviewers as well, yielding a precision or positive predictive value (PPV) of **86.5%** and accuracy of **85.5%**. The sensitivity and specificity were **81.1%** and **89.3%** respectively. Of the remaining 118 trials deemed ineligible by LLM, the human benchmark disagreed with 18 of these evaluations (Table 2). This is comparable to those published by previously proposed pipelines, indicating a strong baseline performance by state of art LLMs prior to pipeline considerations.

### Qualitative analysis: Qualitative Content Analysis of the Reasoning Texts from LLM and Human Evaluators

**Figure 2.**
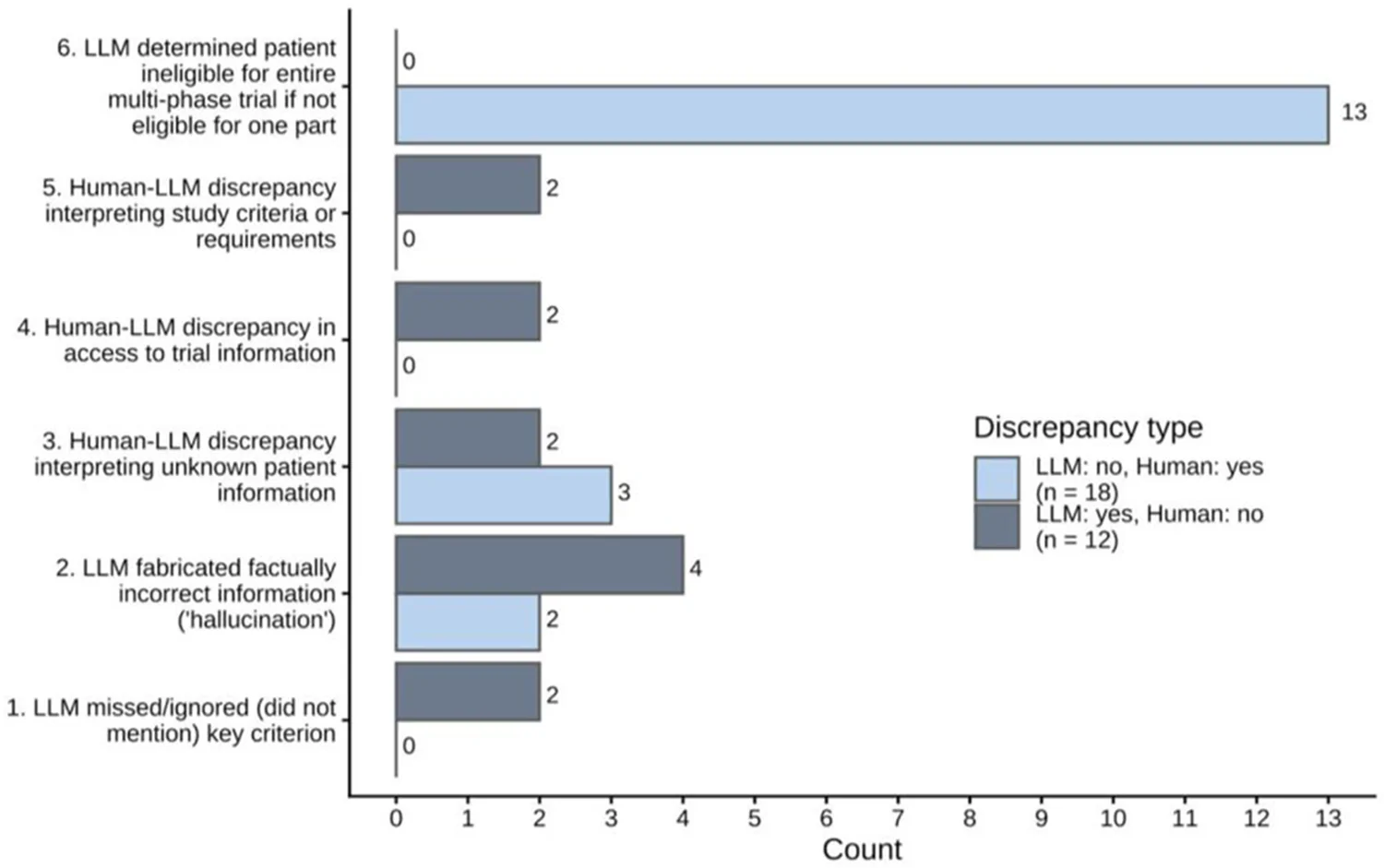
Frequencies of the six error categories, split by type I or type II error

Thirty trials were evaluated differently by the LLM when compared to the human benchmark and categorized as errors. Each was analyzed and categorized into 1 of 6 classes manually by the two human reviewers as described in Figure 2. The most common errors (**22/30; 73.3%**) appeared to be modifiable with either improved prompting [E3 (n = 5), E5 (n = 2), E6 (n = 13): **20/30; 66.7%)**] or more comprehensive trial information access [E4 (n = 2): **2/30; (6.7%)**]. **8/30 (26.7%)** of errors occurred due to LLM “hallucination” (E2, n = 6) or overlooking information (E1, n = 2). With model improvement, we expect these latter error types to become less common. The full LLM and human text along with the researchers’ interpretations are described in Supplementary Materials Table S1 and Table S2.

### Practical Performance

In practice, trials more relevant to the patient’s specific disease and with study locations in cities that meet the patient’s needs would be given priority. For this patient’s case, a preliminary search for trials in the San Francisco Bay Area region of California (composed of nine counties: Alameda, Contra Costa, Marin, Napa, San Francisco, San Mateo, Santa Clara, Solano, and Sonoma) targeting “pancreatic adenocarcinoma (PDAC)” would be prioritized and then extended to metastatic solid tumors to capture a broader set of potentially eligible trials.

Consequently, of the 95 trials determined to be appropriate for our patient by our human benchmark, 29 were in the Bay Area, of which the LLM classified 22 (75.9%) in alignment with the human benchmark. Sixteen were either dedicated to pancreatic cancers (3) or had a pancreatic cancer-specific cohort (13), all correctly classified. 2 trials met both geographic and cancer type needs; both were identified by the LLM:

1. NCT03485209, Efficacy and Safety Study of Tisotumab Vedotin for Patients With Solid Tumors (innovaTV 207): A solid tumor trial with an explicit pancreatic adenocarcinoma cohort. However, this cohort was closed for enrollment at the time of adjudication.
2. NCT05249101, A Study of Ivaltinostate Plus Capecitabine or Capecitabine in Metastatic Pancreatic Adenocarcinoma: A trial specific to advanced pancreatic adenocarcinomas, but our patient was likely only eligible for Phase 1b of this trial. Phase 2 required no progression following initial chemotherapy.

Given these limited results, it would be beneficial to continue and consider “metastatic/advanced solid tumor” trials. Among the 95 trials found in the human benchmark, 8 in the San Francisco Bay Area could be classified as such, of which 7 were found by our process. The one trial that was not found was due to a type 6 error (the LLM dismissed the trial because the patient would have been eligible only for phase 1 and not phase 2).

## DISCUSSION

In this study, we evaluated a simple version of a zero-shot, LLM-assisted clinical trial matching process built upon the ClinicalTrials.gov API to both retrieve and assess the relevance of trials for a given patient from a free-text patient narrative. Our findings from this preliminary real-world use case study contributed to the growing evidence demonstrating that LLMs can meaningfully assist humans in the patient-trial matching task, even without pre-structuring study details (which is often inconsistent or complicated) or requiring access to patient records, which can be time-consuming, unstructured, and potentially limits the flexibility of the match. In addition, the qualitative error analysis categorized typical human-LLM discrepancies in this area, revealing potential blind spots. We found that trial descriptions are often vague, leading to instances where – even for a human – the eligibility of a patient was difficult to conclude. Perhaps as models improve and users become increasingly familiar with prompting techniques, ambiguities due to certain discrepancies can be overcome.

### Comparison to non-LLM-assisted tools

Despite these limitations, an LLM-assisted clinical trial matching process appeared superior to previously existing questionnaire-based or simple algorithm-based tools for identifying relevant trials. To assess this, we compared the performance of our zero-shot LLM-assisted process with traditional trial matching tools, including 1) ClinicalTrials.gov[6]; 2) the Pancreatic Cancer Action Network (PanCAN) Clinical Trial Finder[14]; and 3) Ancora.ai[7] (Table 3). We find that these platforms – often questionnaire- or algorithm-based – miss subtleties in patient characteristics that facilitate better matches.

Ancora.ai identified 59 total trials in California for our patient, of which our human benchmark agreed with 35 (and the LLM agreed with 39). Of these 35 “yes” trials, the LLM found all except two, both due to errors classified by the research team as hallucinations (error type #2). 12/20 of the Ancora.ai-matched trials that the LLM did not approve appeared to have been matched by Ancora.ai due to insufficient mutation information. For example, Ancora.ai’s patient questionnaire asked about KRAS mutations but did not require further specifications. Three such trials (NCT05410145, NCT04956640, and NCT03785249) required a specific *type* of KRAS mutation our patient did not have, revealing the limitations of a questionnaire-based patient-trial matching system.

The Pancreatic Cancer Action Network (PanCAN) Clinical Trial Finder identified only a total of 5 trials for our patient case (due to their focused scope), of which 2 were found to be appropriate by our human benchmark (both were assessed correctly by the LLM). The other 3 trials were deemed ineligible because they required either no progression on first-line therapy, or required specific biomarkers/genetic findings (BRCA1/2 mutation or MSI-high disease) – criteria not met by our use case and not captured in the PanCAN questionnaire. Finally, the ClinicalTrials.gov interface returned 223 trials – far too many for practical assessment or prioritization – and included trials with locations that were no longer recruiting.

We found that LLM-based tools were able to synthesize free-text patient summaries and assess trials using patient features that may be missing from a form or questionnaire, which were rarely comprehensive enough to capture all key details of a patient’s case. Critically, none of the above platforms allowed for specific mutation, immunohistochemistry, or treatment history input.

### Limitations

Our study had several limitations. First, this evaluation was done for a single patient case, precluding broader generalizability. Second, the tested LLM-assisted matching approach decidedly used a zero-shot prompt (to consider ‘base’ performance of LLMs) and relied on the ClinicalTrials.gov search engine to retrieve trials using user-specified conditions instead of a trial retrieval algorithm (for example, like the one used by TrialGPT, which we were unable to access at the time of evaluation). Third, the human benchmark was not reviewed by an oncologist or other specialist. Future work may continue evaluation of LLM-assisted patient-trial matching implementations in real-world clinical settings across disease types and consider testing updated models, prompts, and processes. Finally, while the temperature settings (used to regulate the “randomness” in LLM output) were set to zero in this work for reproducibility, recommended temperature values could be identified. Our preliminary testing in this area demonstrated little change in binary adjudications from temperatures 0 to 0.5, though the LLM’s self-assessed confidence levels were found to vary slightly.

## CONCLUSION

We evaluated a simple zero-shot LLM-assisted patient-trial matching process using a real-world pancreatic patient profile. We believe LLM-assisted patient-trial matching systems could be helpful for patients if integrated into a process including in-the-loop human raters to evaluate LLM assessments. Others have previously demonstrated that substantial human time was saved in such an integrated process[17]. Given our and others’ results, LLM-assisted trial matching processes appeared able to streamline trial identification enough to become a routine, seamless part of metastatic cancer care. We imagine a dynamic, collaborative workflow for patients and oncologists to make informed and up-to-date decisions about clinical trial options. Continued evaluations across diverse metastatic cancer phenotypes are needed to further characterize LLM performance across scenarios.

## Supplementary Material

Supplement 1

## Figures and Tables

**Table 1.** Examples of the six error categories that emerged from qualitative analysis of human and LLM reasoning texts.

Error category	Example (from case study)
**E1**: LLM ignored key criterion	NCT05846659: *“LLM does **not at all acknowledge inclusion requirement** for “‘oligoprogressive’ disease” which human raters use to make their decision.”*
**E2**: LLM fabricated factually incorrect information (“hallucination”)	NCT04626635: *“**LLM states ‘FOLFIRINOX may include PD-1/PD-L1 inhibitors,’ which is false**; FOLFIRINOX may be combined with PD-1/PD-L1 inhibitors but does not contain them.”*
**E3**: Human-LLM discrepancy interpreting unknown patient information	NCT05150691: “*LLM **assumes patient has HER2-expressing tumor due to no mention of it in patient vignette**. Human does not.”*
**E4**: Human-LLM discrepancy in access to trial information	NCT05489211: “*LLM **could not access portion of trial information describing sub cohort eligibility**. If human evaluated using the same information, LLM would be correct*.”
**E5**: Human-LLM discrepancy interpreting study requirements	NCT05123482: “*LLM acknowledges that patient isn’t explicitly eligible for specific sub-studies but does meet overall inclusion criteria. **Human assumed specified sub-studies were the only ones conducted***.”
**E6**: LLM determined patient ineligible for entire multi-phase trial if not eligible for one part	NCT05318573: “*LLM technically correct: patient is not eligible for expansion phase of study. **Human raters were more generous**, as patient could be eligible for the safety run-in (phase I)*.”

**Table 2.** Concordance and Discordance between LLM and Human Trial Evaluations

	Eligible – *LLM Assessment*	Ineligible – *LLM Assessment*	Total
**Eligible** – *Human Assessment*	**77**	**18**	95
**Ineligible** – *Human Assessment*	**12**	**100**	112
Total	89	118	**207**

**Table 3.** Input navigation and performance of select existing tools

Tool	Total Trials matched	Of the trials found, how many did the human evaluator determine eligible?	Patient and trial preference navigation
**Testing process** 08/04/2024	**89**	**77**/89 (86.5%)	
**Ancora.ai** 08/06/2024	**59** (all match levels) in California **5** “Very Good” matches (Ancora.ai’s ranking system was unclear)	**35**/59 (59.3%) (all match levels) in California **3**/5 (60%)	**Dropdown**: Pancreatic Cancer **Location**: within 400mi of [*redacted*], California, United States **Type:** Adenocarcinoma **Stage:** Metastatic **Brain metastases:** I don’t know **Mutations**: BRCA: No; KRAS: Yes **Received treatment**: Yes > **Chemotherapy**: Yes **Surgery**: No **Radiotherapy**: No **Immunotherapy**: No **Sex**: [*redacted*] **Year of birth**: [*redacted*] **Other diseases** (checkboxes): None Which describes your physical ability (**ECOG**): Skipped **Ethnicity**: Skipped **Phase**(s): 2, 3, 4 **Trial type**(s): Interventional **Recruiting status**: Recruiting, not yet recruiting
**Pancreatic Cancer Action Network Clinical Trial Finder** 08/06/2024	**5**	**2**/5 (40%)	**Location**: within 400 miles of [*redacted*], California, United States **Type**: Adenocarcinoma **Stage**: Stage IV **Stage at diagnosis**: Stage IV **Treatment**: Chemotherapy > FOLFIRINOX **Treatment for advanced disease:** All therapies have been for advanced disease **Regimens**: 1 **Phases**: II, III
ClinicalTrials.gov 08/02/2024	**223**	**98**/223 (43.9%)	**Condition**: solid tumors OR pancreatic cancers OR pancreatic Adenocarcinoma **Location**: California **Study Status**: Recruiting and not yet recruiting **Sex**: [*redacted*] **Age**: Adult **Phase**: II, III, IV **Study Type**: Interventional **Phase**(s): 2/3/4 (includes ½ trials)

## Data Availability

The data that support the findings of this study are available from the corresponding author upon reasonable request.
