# Supplementary material for: Assessment of Zero-Shot Large Language Model (LLM) Assisted Clinical Trial Matching Processes: A Metastatic Cancer Use Case
Source: medRxiv. 2026 Jul 10:2026.07.06.26354647. Preprint. [Version 1] doi: 10.64898/2026.07.06.26354647 (PMC13370548; doi:10.64898/2026.07.06.26354647)
Supplement: Supplementary file 1 [file NIHPP2026.07.06.26354647v1-supplement-1.pdf]

medRxiv preprint doi: https://doi.org/10.64898/2026.07.06.26354647; this version posted July 10, 2026. The copyright holder for this preprint
(which was not certified by peer review) is the author/funder, who has granted medRxiv a license to display the preprint in perpetuity.
It is made available under a CC-BY 4.0 International license .

Supplementary Materials

Appendix I. A Zero-shot Prompt for GPT-4 Turbo

You are now a precise and diligent medical research expert. Your task is to determine whether a patient qualifies for a clinical study based on the provided patient information ('detailedInformation') and clinical trial information: study title ('briefTitle'), study summary ('briefSummary'), and eligibility criteria ('eligibilityCriteria'). Please ignore all laboratory-related requirements in 'eligibilityCriteria' when making your decision (ex. AST, creatinine, blood cell counts, etc.).

When formulating your decision, consider the following:
1. Generally, assume the patient is eligible for the trial unless: 1) any details in the patient's information are clearly incompatible with the trial inclusion requirements, or 2) any details in the patient's information clearly meet any of the trial's exclusion criteria.
2. Use the additional information in the title or summary to strengthen your judgement.
3. If a key detail about the patient is missing, assume the patient is compliant but lower the confidence level of your judgment in the 'confidence_level' portion of your response. The more unknowns, the lower your confidence level.

Please evaluate the patient's eligibility for participation in this clinical study and respond in valid JSON format. You must provide all the required fields as described below:
1. 'meet_inclusionCriteria' (string): Please list only the study inclusion criteria that the patient clearly meets based on their provided information.
2. 'not_meet_inclusionCriteria' (string): Please list only the study inclusion criteria that the patient clearly does NOT meet based on their provided information.
3. 'meet_exclusionCriteria' (string): Please list the study exclusion criteria that the patient clearly meets based on their provided information.
4. 'not_meet_brieftitle' (string): Please list any details in the study title (found in "briefTitle") that do not appear to match up with the patient's characteristics.
5. 'not_meet_briefsummary' (string): Please list details in the study summary (found in "briefSummary") that do not appear to match up with the patient's characteristics.
6. 'thought' (string): Based on these evaluations, provide a step-by-step thought process of evaluating this patient's eligibility for the clinical study.
7. 'result' (string): Provide a final judgment: 'yes' if you believe the patient is eligible for this study, or 'no' otherwise.
8. 'confidence_level' (numeric): Finally, provide a numeric value (from 0-1) indicating your confidence in this judgment.

study:
  "eligibilityCriteria": {*eligibilityCriteria*}
  "briefTitle": {*briefTitle*}
  "briefSummary": {*briefSummary*}
patient:
  "detailedInformation": {*patient_info*}
answer:

medRxiv preprint doi: https://doi.org/10.64898/2026.07.06.26354647; this version posted July 10, 2026. The copyright holder for this preprint (which was not certified by peer review) is the author/funder, who has granted medRxiv a license to display the preprint in perpetuity.
It is made available under a CC-BY 4.0 International license .

Appendix II. The User Interface for the tested LLM-assisted Clinical Trial Matching Process

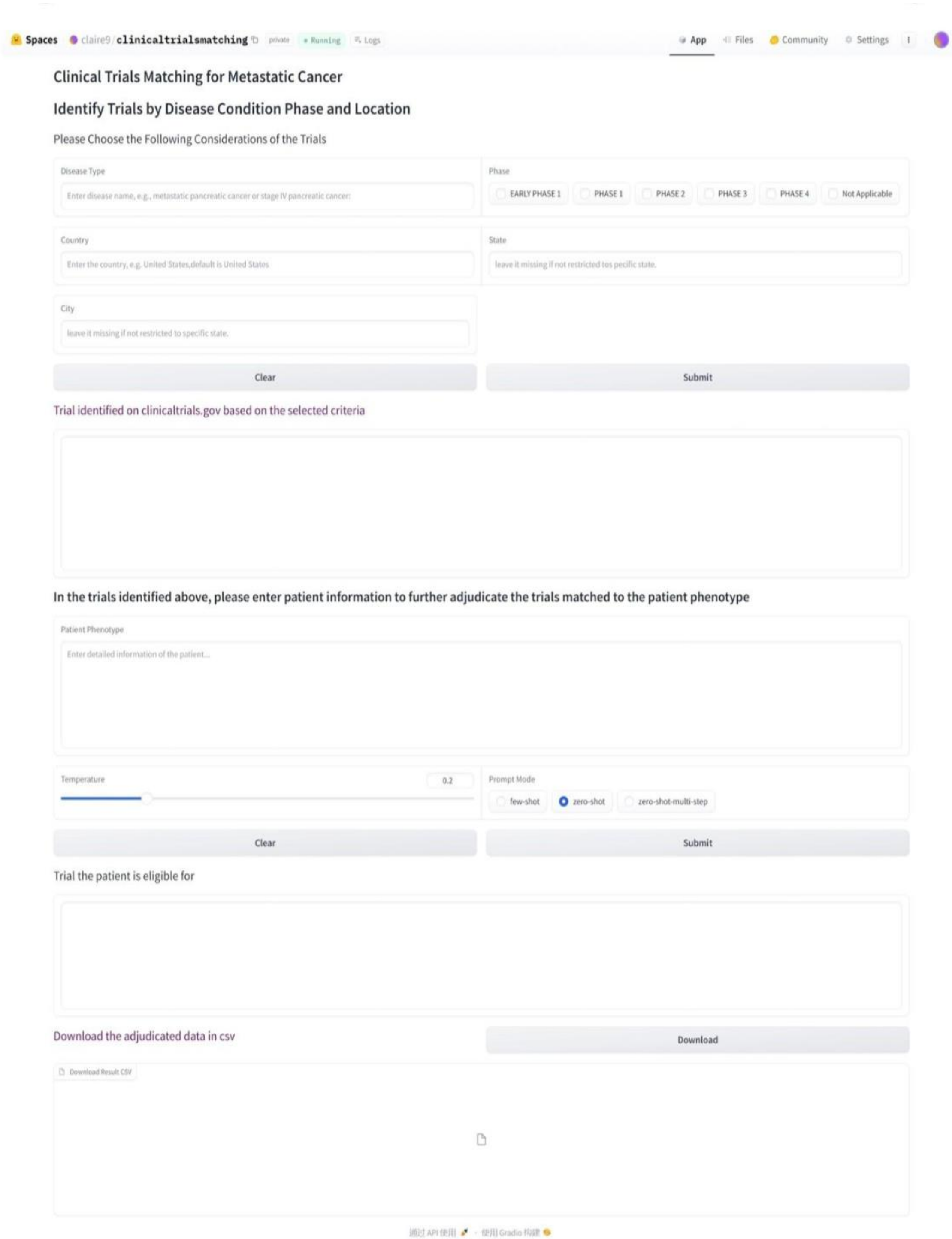

medRxiv preprint doi: https://doi.org/10.64898/2026.07.06.26354647; this version posted July 10, 2026. The copyright holder for this preprint (which was not certified by peer review) is the author/funder, who has granted medRxiv a license to display the preprint in perpetuity.
It is made available under a CC-BY 4.0 International license .

Table S1. LLM "thought process" vs. Human thought process for False Positive discrepancies – LLM: yes, Human: no (n = 12).

NCT ID	LLM "thought"	Human "reason"	Discrepancy Classification	Analysis
NCT05123482	"The patient is a [*redacted age*]-year-old [*redacted sex*] with metastatic pancreatic cancer, which is a solid tumor, thus fitting the study's focus on advanced or metastatic solid malignancies. [*The patient*] meets the age requirement, has measurable disease, and an ECOG status likely between 0-1 given [*their*] ability to comply with study procedures. [*Their*] life expectancy appears to be over 12 weeks as there's no indication otherwise. **[*The patient*] does not fit the specific cancer types listed for the sub-studies but meets the general criteria for the main study.** There are no exclusion criteria met based on the provided information. The study title and summary align with the patient's condition."	"Tumor type doesn't appear eligible for substudies"	**5**) Human-LLM discrepancy in interpreting study criteria or information.	The LLM acknowledges that while the patient isn't explicitly eligible for specific sub-studies, [*the patient*] does meet all key inclusion criteria. The human evaluator assumed specified sub-studies were the only ones conducted.
NCT05150691	"The patient meets several key inclusion criteria such **as having a HER2-expressing advanced solid tumor**, at least one measurable lesion, and willingness to comply with study procedures. However, the patient's ECOG performance status, LVEF, organ functions, and life expectancy are not specified, which are crucial for determining eligibility. No exclusion criteria are clearly met based on the provided information. The study title and summary align with the patient's condition of having an advanced solid tumor."	"HER2 not expressed by tumor"	**3**) Human-LLM discrepancy in interpreting unknown patient information	The LLM assumes patient has an HER2-expressing tumor, while the human does not, due to the rarity of this event. This assumption may be due to the prompt, which encourages erring on the side of inclusion in the case of missing patient information.
NCT06028074	"The patient meets several key inclusion criteria: they have provided written informed consent, have an ECOG performance status likely between 0-1 given their ability to comply with study procedures, and have a confirmed advanced solid tumor (pancreatic ductal adenocarcinoma) that is unresectable and measurable. **They have also failed previous FDA-approved PD-1/PD-L1 inhibitor treatment, which aligns with the study's requirement for subjects who have progressed on such therapies.** There are no exclusion criteria clearly met by the patient based on the provided information. The study title and summary are consistent with the patient's condition of having an advanced solid malignancy."	"Patient has not previously received a PD-1 inhibitor	**2**) LLM "hallucinated" factually incorrect information	The LLM either "makes up" that the patient was previously treated with a PD-1/PD-L1 inhibitor or mistakes FOLFIRINOX for a PD-1/PD-L1 inhibitor (which it is not).
NCT05086692	"The patient is a [*redacted age*]-year-old [*redacted sex*], meeting the age criterion. [*The patient*] has a confirmed metastatic solid tumor (pancreatic ductal adenocarcinoma), which is listed under the conditions for the study. The patient's disease is measurable as per RECIST v1.1, and [*the patient*] has a life expectancy of over 12 weeks. [*The patient*] has expressed willingness to comply with study procedures, fulfilling the consent requirement. There is no information suggesting that the patient meets	"MSI-H pancreatic cancer is the only one mentioned"	**4**) Human-LLM discrepancy in access to trial information	The LLM did not have access to the "Conditions" portion of the ClinicalTrials.gov posting for this trial, but the human rater did. The human rater noticed a specification for MSI-

medRxiv preprint doi: https://doi.org/10.64898/2026.07.06.26354647; this version posted July 10, 2026. The copyright holder for this preprint (which was not certified by peer review) is the author/funder, who has granted medRxiv a license to display the preprint in perpetuity.
It is made available under a CC-BY 4.0 International license .

	any exclusion criteria, such as recent treatments or other medical conditions that would disqualify [*them*]. The study title and summary do not provide specific exclusion details that would apply to this patient."			H pancreatic cancer and rejected this trial.
NCT04423029	"The patient has metastatic pancreatic ductal adenocarcinoma, which is a type of advanced solid tumor. The patient's disease has progressed despite standard therapy (FOLFIRINOX), meeting the inclusion criteria of having an advanced/metastatic solid tumor for which standard therapy has failed. The patient also has clinical evidence of disease, fulfilling another inclusion criterion. There is no information suggesting that the patient meets any exclusion criteria, such as concurrent anticancer treatment or a history of other malignancies within the last 3 years. The study title and summary do not specify tumor types beyond 'advanced solid tumors,' which includes the patient's condition. Therefore, the patient appears to match the study's focus."	"Pancreatic cancer not seemingly included in inclusion criteria"	**1**) LLM missed/ignored (did not mention) key detail	The LLM clearly missed a portion of the inclusion criteria that specifically listed all accepted tumor types, and pancreatic cancer was not one of them. The title, however, only specifies "Advanced Solid Tumors"; the error may have occurred for this reason.
NCT05945823	"The patient is a [*redacted age*]-year-old [*redacted sex*], meeting the age criterion. [*The patient*] has confirmed unresectable and measurable metastatic pancreatic ductal adenocarcinoma, fitting Cohort B's requirements. **The patient has previously failed one regimen of FOLFIRINOX, but no other systemic treatments for metastatic disease, aligning with the inclusion criteria.** The patient's disease is measurable by RECIST guidelines. [*They*] can take medications orally. There are no exclusion criteria met based on the patient's information. The study title and summary align with the patient's condition as they pertain to solid tumors and combination therapy with PD-1 antibody."	"Patient has had prior FOLFIRINOX therapy; study requires no history of prior therapy for pancreatic cancer"	**2**) LLM "hallucinated" factually incorrect information	The study inclusion criteria clearly states a requirement for no prior systemic treatment, which the LLM acknowledges, but then indicates should be okay for this trial. We classify this error as a hallucination due to the clear misinterpretation.
NCT04083599	"The patient is a [*redacted age*]-year-old [*redacted sex*] with confirmed unresectable and measurable metastatic pancreatic ductal adenocarcinoma (PDAC), which aligns with the study's inclusion criteria for subjects **with metastatic PDAC and no prior treatments for metastatic disease.** The patient's age meets the study requirement, and the disease is measurable as per RECIST 1.1. There is no information suggesting that the patient meets any exclusion criteria, such as recent treatments or unresolved toxicities from previous therapies. The patient's characteristics also align with the study's title and summary, which focus on malignant solid tumors and specifically mention metastatic conditions."	"Patient has had prior FOLFIRINOX therapy; study requires no history of prior therapy for pancreatic cancer"	**2**) LLM "hallucinated" factually incorrect information	The study inclusion criteria clearly requires no previous radiotherapy, surgery, chemotherapy, or investigational treatment for metastatic disease; the LLM clearly made up the patient's "no prior treatments"; thus, we classify as a hallucination.
NCT05101070	"The patient meets most of the inclusion criteria: age, type of cancer, measurable disease, willingness to provide samples, and estimated life expectancy. **The patient does not have one of the specific tumor types listed for Part A, but this does not exclude**	"Pancreatic cancer not seemingly included in any of the 3 cohorts"	**5**) Human-LLM discrepancy in interpreting study criteria or information.	The LLM assumed that because specific tumor types were specified for Part A only, Part B and C may have different

medRxiv preprint doi: https://doi.org/10.64898/2026.07.06.26354647; this version posted July 10, 2026. The copyright holder for this preprint (which was not certified by peer review) is the author/funder, who has granted medRxiv a license to display the preprint in perpetuity.
It is made available under a CC-BY 4.0 International license .

	**[*them*] from other parts of the study.** There is no information suggesting that the patient meets any exclusion criteria. The study title and summary are consistent with the patient's condition."			requirements. However, Part B and C appear to select tumor types from the same Part A cohort. This is fairly clear and the LLM misinterpreted it.
NCT05846659	"The patient is a [*redacted age*]-year-old [*redacted sex*] with metastatic pancreatic cancer, which fits the study's requirement for patients with solid tumor malignancies. [*The patient*] has progressed on prior anti-cancer therapy (FOLFIRINOX), aligning with the study's focus on patients who have shown progression after previous treatments. The patient's disease is evaluable per RECIST 1.1, and [*the patient*] has metastatic lesions suitable for radiotherapy, which is necessary for the PULSAR treatment specified in the study. The patient's ECOG performance status is assumed to be 1 since there is no information suggesting otherwise, and [*the patient*] has expressed willingness to comply with study procedures. There is no mention of prior receipt of STING agonists or therapeutic radiotherapy to all progressive lesions, nor is there any indication of uncontrolled illnesses or other exclusion criteria being met."	"There is no indication that patient's disease is oligometastatic or "oligoprogressive"	**1**) LLM missed/ignored (did not mention) key detail	The LLM does not acknowledge the requirement for a "diagnosis of 'oligoprogressive' disease" from the inclusion criteria, which the human raters use to make their decision. As there is no mention of this detail in the reasoning, we assume the LLM missed this detail entirely.
NCT04482309	"The patient has confirmed unresectable and measurable metastatic pancreatic cancer, which aligns with the inclusion criteria for Cohort 6 in Part 1 of the study. The patient has progressed on prior treatment (FOLFIRINOX), meeting another inclusion criterion. There is no information suggesting that the patient meets any exclusion criteria, such as having conditions listed in the exclusion criteria or a primary diagnosis that would exclude them from the study. The study title and summary both align with the patient's condition as they focus on **HER2-expressing tumors and the patient's type of cancer is included in the study cohorts**."	"HER2 not expressed by tumor"	**3**) Human-LLM discrepancy in interpreting unknown patient information	The LLM automatically assumes the patient has an HER2-expressing tumor, while this is not specified in the prompt. The human does not, due to the rarity of this event. Again, this may be due to the prompt encouraging the LLM to err on the side of inclusion.
NCT05489211	"The patient is a [*redacted age*]-year-old [*redacted sex*], meeting the age and gender inclusion criteria. [*The patient*] has a documented advanced metastatic malignancy (pancreatic ductal adenocarcinoma), which aligns with the study's focus on advanced/metastatic solid tumors. The patient has at least one measurable lesion, fulfilling another inclusion criterion. [*The patient*] has expressed willingness to comply with study procedures, indicating capability of giving informed consent. There is no information suggesting that the patient meets any exclusion criteria, such as significant comorbidities or conditions listed in the exclusion criteria. The study title and summary both emphasize treatment for advanced solid tumors, which matches the patient's condition."	"Pancreatic cancer doesn't appear to be in the list of substudies"	**4**) Human-LLM discrepancy in access to trial information	The LLM did not have access to the "Detailed Description" portion of the trial information which describes the eligible condition types for each subcohort. If human evaluation was done on just the eligibility criteria, the LLM is correct.

medRxiv preprint doi: https://doi.org/10.64898/2026.07.06.26354647; this version posted July 10, 2026. The copyright holder for this preprint (which was not certified by peer review) is the author/funder, who has granted medRxiv a license to display the preprint in perpetuity.
It is made available under a CC-BY 4.0 International license .

NCT01954992	"The patient is a [*redacted age*]-year-old [*redacted sex*] with confirmed metastatic pancreatic cancer, which aligns with the study's requirement for participants with metastatic pancreatic cancer who have failed gemcitabine-based therapy. The patient has only received one prior systemic therapy regimen (FOLFIRINOX), which is within the study's limits. The patient's ECOG performance status is assumed to be 0-1 based on the absence of any mention of significant functional limitations. The patient has expressed willingness to comply with study procedures, meeting the consent requirement. There are no indications from the provided information that the patient meets any exclusion criteria."	"Patient has not received gemcitabine-containing treatment; study requires patients to have previously progressed on gemcitabine treatment."	**2**) LLM "hallucinated" factually incorrect information	The tool appeared to misunderstand the trial's requirement for previous progression on a gemcitabine-based treatment, stating that the patient's previous FOLFIRINOX therapy is suitable. We categorize this as a hallucination due to the clear

medRxiv preprint doi: https://doi.org/10.64898/2026.07.06.26354647; this version posted July 10, 2026. The copyright holder for this preprint (which was not certified by peer review) is the author/funder, who has granted medRxiv a license to display the preprint in perpetuity.
It is made available under a CC-BY 4.0 International license .

Table S2. LLM "thought process" vs. Human thought process for False Negative discrepancies – LLM: no, Human: yes (n = 18).

NCT ID	LLM "thought"	Human "reason"	Discrepancy Classification	Analysis
NCT05318573	"The patient meets several inclusion criteria such as age, consent, having advanced metastatic solid tumors, measurable disease, appropriate performance status, and life expectancy. However, **the study specifically targets urothelial and NSCLC cancers in its expansion phase, which does not align with the patient's pancreatic cancer diagnosis**. The patient does not meet any exclusion criteria explicitly. The primary concern is the mismatch between the patient's cancer type and the study's focus."	"Eligible only for the safety run-in portion; not eligible for expansion."	**6**) LLM determined patient ineligible for entire multi-phase trial if not eligible for expansion / phase II / specific cohort	The LLM is technically correct; the patient is not eligible for the expansion phase of this study. The human raters were more generous with their rating, since the patient could be eligible for the safety run-in.
NCT06425926	"The patient meets several key inclusion criteria such as having a confirmed metastatic solid tumor, progression on standard therapy, and measurable disease. However, **the patient does not meet the Phase 2 specific inclusion criteria for cutaneous melanoma and anti-PD-1 therapy**, which are specific to the study's phase 2 segment. No exclusion criteria are met based on the provided patient information. The study title and summary are consistent with the patient's condition of having an advanced solid tumor."	"Eligible only for phase 1"	**6**) LLM determined patient ineligible for entire multi-phase trial if not eligible for expansion / phase II / specific cohort	The LLM is technically correct; the patient is not eligible for phase 2. The human raters were more generous with their rating, since the patient is eligible for phase 1.
NCT04829539	"The patient meets several inclusion criteria such as age, cancer diagnosis, ongoing cancer treatment, and ability to communicate in English. However, **there is no information provided about the patient's sleep disturbance** score or ECOG performance status, which are crucial for determining eligibility. No exclusion criteria are met based on the available information. The study focuses on cancer-related sleep problems, and the patient's cancer status aligns with the study's target demographic."	"Eligible for trial, but is irrelevant; not a treatment trial and intended for those with sleep problems."	**3**) Human-LLM discrepancy in interpreting unknown patient information	The human rater accepted this trial even though it does not focus on treatment and there is no evidence of sleep disturbance among the patient. The LLM interpreted
NCT06399757	"The patient is [*redacted age*] years old, meeting the age criterion. [*The patient*] has a confirmed diagnosis of metastatic pancreatic ductal adenocarcinoma, which is included in the Phase 1 sub-studies. The patient has expressed willingness to comply with the study requirements. However, the patient does not meet the criterion of having exhausted all standard care therapies, as [*the patient*] has only received one regimen (FOLFIRINOX) and no other treatments. There are no exclusion criteria met by the patient based on the provided information. The study title and summary are consistent with the patient's condition."	"Patient eligible for phase 1 only"	**3**) Human-LLM discrepancy in interpreting unknown patient information	The LLM understands the patient's single previous treatment as evidence that there may be other standard of care treatments available for [*them*], which would make [*them*] ineligible for this trial. The human assumes that the patient must be searching for a trial only after exhausting options.

medRxiv preprint doi: https://doi.org/10.64898/2026.07.06.26354647; this version posted July 10, 2026. The copyright holder for this preprint (which was not certified by peer review) is the author/funder, who has granted medRxiv a license to display the preprint in perpetuity.
It is made available under a CC-BY 4.0 International license .

NCT04440735	"The patient meets the general inclusion criteria for advanced solid tumors with measurable disease and an ECOG performance status likely between 0 and 1, as [*the patient*] is able to comply with study procedures. **However, the specific cohorts mentioned in the study (non-small cell lung cancer and colorectal cancer) do not include pancreatic cancer, which is the patient's diagnosis**. No exclusion criteria are met by the patient's provided information. The study title and summary do not explicitly include pancreatic cancer, which is a key consideration."	"Eligible for part 1 only"	**6**) LLM determined patient ineligible for entire multi-phase trial if not eligible for expansion / phase II / specific cohort	LLM acknowledges patient eligible for part 1 but not part 2; the human accepts this, but the LLM does not.
NCT06191796	"The patient has pancreatic ductal adenocarcinoma, which does not match the study's focus on renal cell carcinoma (ccRCC) or the specific mention of other solid tumors in the study summary. The **inclusion criteria specifically mention clear cell renal cell carcinoma, which the patient does not have**. Therefore, despite the patient's willingness and ability to comply with study procedures, they do not meet the primary disease criteria required for the study."	"Eligible; not the focus"	**6**) LLM determined patient ineligible for entire multi-phase trial if not eligible for expansion / phase II / specific cohort	While the LLM is correct and it is true that the study is aimed at understanding renal cell carcinoma, participants with all advanced unresectable cancers are eligible; the LLM did not acknowledge this.
NCT04626635	"The patient meets several key inclusion criteria: confirmed cancer diagnosis, willingness to provide biopsy samples, and a life expectancy of at least 3 months. However, the patient does not meet the criterion of being PD-1/PD-L1 naive, as they have previously failed a regimen of **FOLFIRINOX, which may include PD-1/PD-L1 inhibitors**. No exclusion criteria are clearly met based on the provided information. The study title and summary do not present any clear mismatches with the patient's characteristics."	"Eligible for expansion cohorts (PD-1 naive)"	**2**) LLM "hallucinated" factually incorrect information	The LLM states that FOLFIRINOX may include PD-1/PD-L1 inhibitors. This is generally false; FOLFIRINOX may be combined with PD-1 inhibitors, but it does not contain them. This is a clear hallucination.
NCT03526835	"The patient meets several inclusion criteria such as having a confirmed solid tumor with metastatic disease, measurable disease, life expectancy over 12 weeks, and is amenable for biopsy. However, the patient's information lacks details on ECOG status, LVEF, and whether a fresh tumor sample is available, which are required by the study. The patient does not meet any exclusion criteria based on the provided information. The **study's focus in the summary on head and neck cancer and mCRC does not align with the patient's pancreatic cancer diagnosis**, which may suggest that this study is not targeting pancreatic cancer specifically."	"Eligible for phase 1, but stopped recruiting. Would have been eligible for expansion if not closed"	**6**) LLM determined patient ineligible for entire multi-phase trial if not eligible for expansion / phase II / specific cohort	While the LLM is correct and the study is indeed heavily aimed at head and neck cancers, the single-agent cohort accepts patients of other tumor types as long as they had been previously treated with 1-2 lines of standard therapy.
NCT03830918	"The patient meets the basic inclusion criteria of having an advanced solid malignancy and being willing to comply with study requirements. However, the specific details in the study title and summary	"Eligible for phase 1 only; perhaps eligible for non-randomized portion of phase 2."	**6**) LLM determined patient ineligible for entire multi-phase trial if not eligible for	While the LLM is correct and the study is focused on small cell lung cancer, the patient can participate

medRxiv preprint doi: https://doi.org/10.64898/2026.07.06.26354647; this version posted July 10, 2026. The copyright holder for this preprint (which was not certified by peer review) is the author/funder, who has granted medRxiv a license to display the preprint in perpetuity.
It is made available under a CC-BY 4.0 International license .

	indicate that the **trial is focused on patients with extensive-stage small cell lung cancer, which does not match the patient's condition of pancreatic ductal adenocarcinoma**. Additionally, the patient's tumor type is not one for which atezolizumab is FDA approved, as required for the Phase 1b, Part 2 cohort."		expansion / phase II / specific cohort	as a general advanced solid tumor.
NCT03425279	"The patient meets several inclusion criteria such as having measurable disease, being over the age of 12, having an ECOG performance status of 0 or 1, and a life expectancy of at least three months. However, the patient's information lacks details on renal, liver, and hematological function, which are necessary to fully meet the inclusion criteria. The patient does not meet any of the exclusion criteria based on the provided information**.** The study title and summary **suggest the study is focused on sarcoma and solid tumors, which does not directly align with the patient's diagnosis of pancreatic ductal adenocarcinoma**. This misalignment raises concerns about the patient's suitability for the study."	"Eligible for phase 1, but recruiting is over"	**6**) LLM determined patient ineligible for entire multi-phase trial if not eligible for expansion / phase II / specific cohort	While the LLM is correct and the study is focused on sarcomas, the patient would have been able to participate as an advanced solid tumor participant in phase 1.
NCT05101356	"The patient meets several inclusion criteria such as age, type of cancer, stage of cancer, and willingness to participate. However, the **patient does not meet specific criteria for Phase 2, Cohort A, as [*their*] cancer is pancreatic, not lung adenocarcinoma, and does not involve labyrinthin**. No exclusion criteria are met based on the provided information. The study's focus on labyrinthin in the summary does not match the patient's tumor characteristics, which could affect the treatment's relevance or effectiveness for this patient."	"Not eligible for phase 2 expansion"	**6**) LLM determined patient ineligible for entire multi-phase trial if not eligible for expansion / phase II / specific cohort	The LLM determined patient wasn't eligible based on the eligibility criteria for a single cohort in phase 2. In addition, the LLM assumes the patient's disease doesn't involve labyrinthin, when this isn't specified. We could consider that a hallucination as well.
NCT03504488	"The patient meets several key inclusion criteria such as having a confirmed advanced solid tumor, failure of standard therapy, measurable disease, and being over 18 years old. However, the **patient does not meet the specific cancer types listed in the dose expansion phase of the study** (NSCLC, TNBC, STS) nor the types mentioned in the study title (TNBC, Head & Neck Cancer, NSCLC, Melanoma). No exclusion criteria are met by the patient. The **study's title and the specific cancer types in the dose expansion phase do not align with the patient's type of cancer, which is pancreatic**."	"The title implies that phase 1 is limited to a selected non-pancreatic tumors; however, the inclusion criteria specify this requirement is only for dose expansion phase."	**6**) LLM determined patient ineligible for entire multi-phase trial if not eligible for expansion / phase II / specific cohort	The LLM determined that because the patient doesn't have the tumor types specified in the dose expansion portion of the study (or mentioned in the title), [*the patient*] isn't eligible.
NCT03157128	"The patient has a metastatic solid tumor (pancreatic ductal adenocarcinoma) that has progressed on standard therapy (FOLFIRINOX), meeting one of the key inclusion criteria. However, the **patient does not have a RET gene alteration, which is a requirement in later phases of the study**.	"Will not be eligible for phase 2; only phase 1"	**6**) LLM determined patient ineligible for entire multi-phase trial if not eligible for expansion / phase II / specific cohort	While the patient does not have the gene alteration that is the primary focus of this study, [*they*] should be eligible for phase 1, which the

medRxiv preprint doi: https://doi.org/10.64898/2026.07.06.26354647; this version posted July 10, 2026. The copyright holder for this preprint (which was not certified by peer review) is the author/funder, who has granted medRxiv a license to display the preprint in perpetuity.
It is made available under a CC-BY 4.0 International license .

	The study title and summary emphasize RET fusion-positive tumors and medullary thyroid cancer, which do not match the patient's tumor type or genetic profile. Therefore, while the patient meets some initial inclusion criteria, they **do not align with the specific focus on RET alterations as the study progresses**."			LLM did not agree with.
NCT04180371	"The patient meets several general inclusion criteria such as age, willingness to comply, and having measurable disease. However, the patient's type of cancer (pancreatic cancer) is not listed among the specific cancers mentioned in the study summary for which the drug's efficacy will be studied. Additionally, the study focuses on tumors with high EphA2 expression, and there is no information provided about the EphA2 status of the patient's tumor. Therefore, despite meeting some inclusion criteria, the patient does not fit the specific cancer types or the EphA2 expression focus of the study."	"Pancreatic cancer can be Epha2+; may be possible if tissue is available."	**3**) Human-LLM discrepancy in interpreting unknown patient information	While the human raters interpret the lack of patient information on EphA2 status with caution, the LLM determines that the omission indicates a lack of mutation.
NCT05888532	"The **patient does not meet the specific histological criteria for any of the cohorts (A, B, C) as [*their*] cancer type (pancreatic ductal adenocarcinoma) is not listed**. The study's **focus, as per the brief summary, is on genitourinary malignancies, which does not align with the patient's condition**. However, the patient meets the age requirement and is willing and able to comply with study procedures. There are no exclusion criteria met by the patient."	"Only cohort A; not really worth it."	**6**) LLM determined patient ineligible for entire multi-phase trial if not eligible for expansion / phase II / specific cohort	Oddly, the LLM states clearly that patient isn't histologically eligible for Cohort A, despite the requirement being "Histologically-confirmed metastatic solid tumor malignancy." For this reason, we believe this error can also be classified as a hallucination.
NCT05756907	"The patient is a [*redacted age*]-year-old [*redacted sex*], which meets the age criterion. [*The patient*] has confirmed metastatic pancreatic cancer, which is a solid tumor, but the study specifically requires PROC (platinum-resistant ovarian cancer) in Part 2, which [*the patient*] does not have. The patient's willingness to comply with study procedures meets another inclusion criterion. However, the study title and summary **clearly indicate the study is focused on ovarian cancer, specifically platinum-resistant, which does not match the patient's condition of pancreatic cancer**."	"Ovarian cancer trial; but eligible for part 1"	**6**) LLM determined patient ineligible for entire multi-phase trial if not eligible for expansion / phase II / specific cohort	The LLM determined that because the patient doesn't have PROC, which the trial is focused on, the patient is ineligible, even though the patient should be eligible for part 1.
NCT05497453	"The patient meets the age requirement and has progressed on a prior systemic therapy, which aligns with some of the inclusion criteria. However, **the patient's type of cancer (pancreatic ductal adenocarcinoma) does not match the study's focus on hepatocellular carcinoma and other solid tumors associated with the MYC oncogene**. Additionally, the patient does not meet several specific inclusion criteria related to hepatocellular carcinoma	"Part 1 only"	**6**) LLM determined patient ineligible for entire multi-phase trial if not eligible for expansion / phase II / specific cohort	The human rater determined that PDAC could be associated with the MYC oncogene, which the LLM did not acknowledge. This could be considered a hallucination, though the patient should be

medRxiv preprint doi: https://doi.org/10.64898/2026.07.06.26354647; this version posted July 10, 2026. The copyright holder for this preprint (which was not certified by peer review) is the author/funder, who has granted medRxiv a license to display the preprint in perpetuity.
It is made available under a CC-BY 4.0 International license .

	and hepatitis B. The patient does not meet any exclusion criteria explicitly."			able to participate in part 1.
NCT06305247	"The patient is a [*redacted age*]-year-old [*redacted sex*], meeting the age criterion. [*The patient*] has a confirmed diagnosis of metastatic pancreatic ductal adenocarcinoma (PDAC), which is listed among the specific cancers in the inclusion criteria. The disease is measurable as per RECIST 1.1, and the patient has expressed willingness to comply with study procedures, including biopsy if needed. **However, the patient's tumor does not harbor the specific MAPKm genetic mutations required by the study**. No exclusion criteria are clearly met based on the provided patient information."	"KRAS is a MAPKm mutation"	**2**) LLM "hallucinated" factually incorrect information	The LLM doesn't acknowledge the patient's KRAS mutation, which could be considered a MAPKm mutation.
